# In Memoriam: Shur-Tzu (Su) Chen, a pioneer in tumor suppressor WWOX for neuroscience

**DOI:** 10.1186/s12929-015-0148-9

**Published:** 2015-06-09

**Authors:** Nan-Shan Chang

**Affiliations:** Institute of Molecular Medicine, College of Medicine, National Cheng Kung University, 1 University Road, Tainan 70101, Taiwan, Republic of China

Professor Shur-Tzu (Su) Chen (1955–2015), who was a pioneer working on tumor suppressor WWOX in neuroscience, died February 28 of lymphoma in Tainan, Taiwan. She was 59 and was survived by her husband, two daughters and a grandson.

Su was a native of Taiwan. She received her Bachelor in Biology at the National Taiwan Normal University in Taipei in 1978, Master in Neuroendocrine at the Imperial College London in 1992, and Ph.D. in Neuroscience at the Charing Cross and Westminster Medical School, University of London, in 1994. She became an Associate Professor at the Department of Cell Biology and Anatomy of the National Cheng Kung University (NCKU), Tainan, Taiwan, in 1995. She chaired the Department from 2000 to 2003. In December 2006, Su was diagnosed to have lymphoma. In 2007, she was promoted to full Professor, and retired a few months later. Su bravely fought her disease for 8 years, undergoing several rounds of chemotherapies, remission and relapse. Even amidst her struggle, Su was determined to live  her life to the fullest, gardening, traveling and performing extensive volunteer work. She journeyed across the globe, visiting over twenty countries in  Asia, Europe, Oceania, Northern/Southern America. She volunteered in the NCKU hospital, guiding foreign patients and teaching histology to medical students. In the past few months of her life, she continued to volunteer in the newly established Chi-Mei Museum in Tainan. Despite her cancer relapse, she continued to entertain museum guests with her knowledge and vision of the arts. Her warmth and charm permanently improved the lives of those who were fortunate enough to meet her. 

Su was my classmate in college from 1974 to 1978, and we have remained good friends ever since.  Su led a colorful and happy life, forming many strong relationships with her friends, colleagues and students. “During the college years, Su used to sit in the front of the classroom and paid her great attention to what teachers had taught us, especially... in the Anatomy course”, says classmate Mr. Chien Wang, now a professional writer. Su had a particular zeal in the science of anatomy. Indeed, she became a Teaching Assistant for Anatomy course in the NCKU Medical College during 1990–1992. In addition, Su loved arts and music. As far as I recall, she loved the songs of Elvis Presley in college. She painted elegantly in both Chinese and Western styles.

She was the leader of our Biology class during our senior year in 1978. Classmate Dr. Weise Chang at the National Institute of Health, USA, put it, “When she decided to run for the class head during our senior year at college her campaign motto was "being a family", which we took to heart, even thirty years later.  Su was never flashy; she wasn't a flag bearer or a chearleader.  Quite on the contrary, she took  a low-key approach, getting what she wanted but being the last to claim credit for her effors. Undoubtedly that persistaence and passion spurred many of her achievements ove the years.”

 That same year, Su met her boyfriend, JP Wang, who was the leader of another Biology class. This couple has undeniable chemistry and married after graduation from college, bringing joy to each other and to us, their friends and classmates. Su’s husband is now a Professor  in the Life Science Department of NCKU. He is noted for his outstanding efforts  in the conservation of  dolphins and whales ("Whale explodes in Taiwanese city". *BBC News*. January 29, 2004).

 Su approached the tumor suppressor WWOX from the perspective of neuroscience, when scientists in the field focused  on the role of WWOX in cancer suppression and gene alterations. In 2000, my group, together with two others, had independently first cloned the tumor suppressor *WWOX* gene at the Guthrie Research Institute, Sayre, Pennsylvania, USA. This gene is located on a common fragile site on chromosome 16q *FRA16D*. Frequent alterations such as gene deletion have frequently been seen in many types of cancer cells. WWOX exerts tumor suppression by blocking cell growth and causing cell death or apoptosis. Su invited me to give a lecture regarding WWOX at the NCKU Medical College on January 2, 2002. Since then, she started expanding her research to investigate the role of WWOX in the developing neural system. To foster a stronger collaboration, Su worked  in my laboratory at the Guthrie Research Institute from April to July in 2004. I recalled that April was particularly snowy and chilly that year, yet Su still rode her bike for shopping and  exploring the small Pennsylvania town where she lived and worked.

Su’s first publication in the Neuroscience journal clearly demonstrated the differential expression of WWOX protein in the developing brain and spinal cord in the embryos and newborns of mice (Neuroscience 124: 831, 2004). The histological details provide the first convincing evidence that WWOX participates in the neural development. One year later, Su had another publication regarding constant light-induced neurodegeneration in the rat eyes (Neuroscience 130: 397, 2005). In our original background work, we demonstrated that when cells are subjected to stress from their surrounding, WWOX becomes activated (via phosphorylation) and relocates from the cytoplasm to the mitochondria and nuclei. As a consequence, the cells expressing activated WWOX start to die or undergo apoptosis. When rats live under constant low-intensity light (500 lux) without a daily darkness cycle for three months, their retinas start to degenerate as the entire cell layers shrink and many neurons undergo apoptosis. For example, substantial death of photoreceptors or neurons occurs, and  increased numbers of activated microglia, astrocytes and Muller glial cells are found  in the damaged outer retina. Su utilized electron microscopy and had shown the presence of activated WWOX in the nuclei and mitochondria of dying neurons. Over the next  few years, Su utilized her outstanding skill in electron microscopy to assist us in elucidating WWOX intracellular translocation in cells (Clin Cancer Res 11: 5769, 2005; J Biol Chem 284: 16049, 2009; Cell Death Dis 4: e792, 2013; Anal Cell Pathol (Amst) 36: 133, 2013). I was amazed at her findings regarding transforming growth factor beta (TGF-β)-induced relocation of WWOX and hyaluronidase Hyal-2 to the nucleus (J Biol Chem 284: 16049, 2009). This study delineates  a novel signal pathway for TGF-β/Hyal-2/WWOX and the downstream Smad proteins for promoter activation and cell growth and death.

Su also conducted ongoing studies on  Parkinson's disease (PD)-like symptoms. Rats received dopaminergic neurotoxin 1-methyl-4-phenyl-pyridinium (MPP+) by injection to the striatum and cortex (Eur J Neurosci 27: 1634, 2008). She showed MPP + −induced neurodegeneration is due in part to the activation of WWOX. Most importantly, a short Tyr33-phosphorylated WWOX peptide with only 11 amino acid residues was made and this peptide effectively blocked MPP + −induced neuronal death in the rat brains, making it a potential therapeutic candidate. On December 4, 2008, I was invited to give a seminar at the premier neuroscience center, New York State Institute for Basic Research in Developmental Disabilities at Staten Island. After my talk, scientists in the Institute stated their appreciation for the outstanding immunoelectron microscopy  skills we had in Taiwan. I credited the work for Su’s expertise in obtaining and processing our high-resoultion electron microscope images.  

In one of her most noteworthy work in neural damage, Su reported that upon sciatic nerve transection in rats, injured neurons in the dorsal root ganglia rapidly exhibit accumulation of activated WWOX and many transcription factors such as CREB, c-Jun, NF-κB and ATF3 in the nuclei within hours or the first week of injury (PLoS One 4: e7820, 2009). The continual nuclear accumulation of these proteins ultimately leads to neuronal apoptosis. WWOX physically interacts many transcription factors. *In vitro* experiments revealed that WWOX blocks the transcription function of certain transcription factors and yet enhances others. In a physiological sense, activated WWOX works synergistically with some transcription factors to cause neuronal death, whereas antagonizes others to nullify each other’s function so that neurons can survive.

 Most recent studies from clinical findings show that when *WWOX* gene undergoes homozygous mutations at specific bases, patients are WWOX-deficient  in the body, resulting in  growth retardation, small brains, ataxia, epilepsy, mental retardation, and early death (Biochim Biophys Acta 1846: 188, 2014; Oncotarget 5: 11792, 2014; review articles). Similar findings have also been shown in mice and rats. That is, when one loses WWOX in the body, the person will die at a very early age from neural and metabolic disorders. Furthermore, when WWOX levels decrease with age, neurodegenerative diseases such as Alzheimer’s disease manifest  (J Biol Chem 279: 30498, 2004; Oncotarget 5: 11792, 2014; Oncotarget 6: 3578, 2015).

Clearly, Su had pioneered her work in the WWOX field for neuroscience. Conceptually, WWOX is needed for the development of neural system in the embryos and newborns. Without WWOX, newborns will suffer neural disorders, growth retardation and death. During neural damage, WWOX works together with critical transcription factors to remove dying neurons.

 As a researcher in the field of tumor suppressors,  Su was unfortunately  taken away by the devastating lymphoma. Still, I remember her faithful friendship, exceptional kindness and joyful presence, even as she endured chemotherapy and bravely faced her own mortality. Su was baptized on January 1, 2015, two months before her passing. Su is now a beautiful angel resting in heavenly peace. Su Chen possessed a passion for life, a curious, deft intellect and an unceasing perseverance that drove her considerable achievements in her time on this earth. She leaves a great legacy for the scientific community to follow. 

